# Epigastric Pain Caused by Left Upper Quadrant Acute Appendicitis in the Setting of Undiagnosed Intestinal Malrotation

**DOI:** 10.7759/cureus.102446

**Published:** 2026-01-27

**Authors:** Saher Bhati, Sundareswaran Sharma, Fatma Abdulle, Syed Mohammad Javed

**Affiliations:** 1 General Surgery, Mohammed Bin Rashid University of Medicine and Health Sciences, Dubai, ARE; 2 General Surgery, Dubai Health, Dubai, ARE

**Keywords:** abdominal pain, acute appendicitis, adult congenital anomaly, appendectomy variants, emergency surgery, epigastric, epigastric pain, intestinal malrotation, left-sided appendicitis, sepsis

## Abstract

Acute appendicitis is a common surgical emergency that may present atypically due to anatomical variations. We report a case of a previously healthy male in his forties presenting with epigastric abdominal pain, vomiting, and fever, initially managed as gastritis. Contrast-enhanced computed tomography (CT) of the abdomen revealed acute appendicitis in the left upper quadrant on a background of previously undiagnosed intestinal malrotation. The patient underwent emergency laparoscopic appendectomy, with intensive perioperative care for septic shock. He ultimately made a full recovery. This case highlights the importance of maintaining a high index of suspicion for acute appendicitis in adults presenting with epigastric pain and illustrates the role of early CT in identifying atypical presentations caused by underlying anatomical variants.

## Introduction

Acute appendicitis is one of the leading causes of acute abdominal pain globally [1]. It typically presents as periumbilical pain that migrates to the right lower quadrant and is often accompanied by fever, anorexia, nausea, and vomiting [2]. However, the classical pain sequence is present in only about 50% to 60% of cases [3]. Atypical presentations may occur due to variations in the anatomical position of the appendix [4], leading to delayed diagnosis, increased risk for perioperative complications, prolonged surgery, and longer hospital stays [5,6].

Intestinal malrotation is a congenital anomaly characterized by abnormal rotation and fixation of the midgut during embryological development, leading to atypical positioning of the small and large intestines and their mesenteric attachments [7]. While most cases are diagnosed in childhood, some remain undetected until later in life [8,9]. In adults, the prevalence is low, with CT colonography studies reporting a prevalence of 0.17% to 0.75% [10]. Many adults are asymptomatic or present with vague gastrointestinal symptoms and are incidentally diagnosed on imaging [11,12]. Altered appendiceal position in intestinal malrotation can result in atypical presentations of appendicitis, with upper or left-sided abdominal pain, mimicking other pathologies [13,14].

We report a case of previously undiagnosed intestinal malrotation presenting with epigastric abdominal pain secondary to acute appendicitis, highlighting the diagnostic challenges posed by undiagnosed anatomical variations in adults.

## Case presentation

Patient presentation

A previously healthy man in his forties presented to the emergency department with a two-day history of progressively worsening epigastric abdominal pain. The pain was stabbing in nature, initially localized to the epigastrium and later radiated toward the periumbilical region. It was associated with anorexia, nausea, three episodes of non-bilious emesis, and a one-day history of fever (39°C). He reported no bowel movements for two days but was passing flatus. The pain was unrelieved by over-the-counter analgesia. He denied any other symptoms.

One day before the presentation, he sought medical attention at a primary care clinic for his abdominal pain and vomiting. He was treated for presumed gastritis with analgesia and a proton pump inhibitor. However, his symptoms persisted without improvement.

He had no past medical or surgical history and no known congenital anomalies. He did not take any chronic medications and had no known allergies. There was no known family history of medical conditions. He denied alcohol use, smoking, or recreational drug use.

On examination, he appeared uncomfortable but was alert. Vital signs showed fever (39°C), tachycardia (116 beats per minute), hypotension (blood pressure 91/58 mmHg), a respiratory rate of 18 breaths per minute, and oxygen saturation of 99% on room air. Abdominal inspection revealed no prior surgical scars. His abdomen was soft, non-distended, with marked tenderness localized to the epigastric and periumbilical regions. There was no guarding, rebound tenderness, or rigidity. Murphy's sign, Rovsing's sign, the psoas sign, and the obturator sign were all negative. Bowel sounds were normoactive. There were no palpable abdominal masses or organomegaly. Examination of the respiratory and cardiovascular systems was unremarkable.

Investigations

Initial investigations revealed leukocytosis (17.8 × 10⁹/L) with an elevated C-reactive protein (CRP) of 82.1 mg/L. Serum electrolytes, renal function, and liver function tests were within normal limits. Plasma lactate was elevated at 3.48 mmol/L. Urinalysis was not suggestive of infection, and urine culture results showed no growth. Blood cultures obtained before antibiotic administration grew extended-spectrum beta-lactamase-producing *Escherichia coli*.

**Figure 1:**
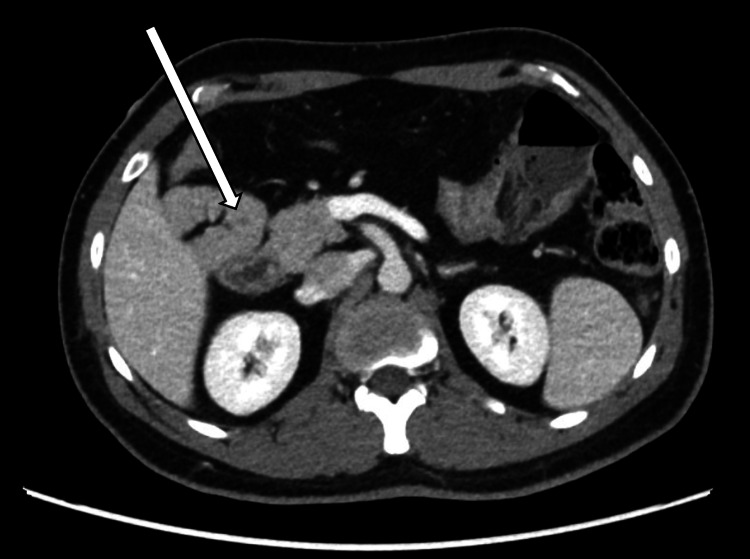
Failure of the duodenojejunal junction to cross the midline Axial CT image demonstrating the duodenojejunal flexure, which fails to cross the midline and is located on the right side of the abdomen, consistent with intestinal malrotation. CT, computed tomography.

**Figure 2:**
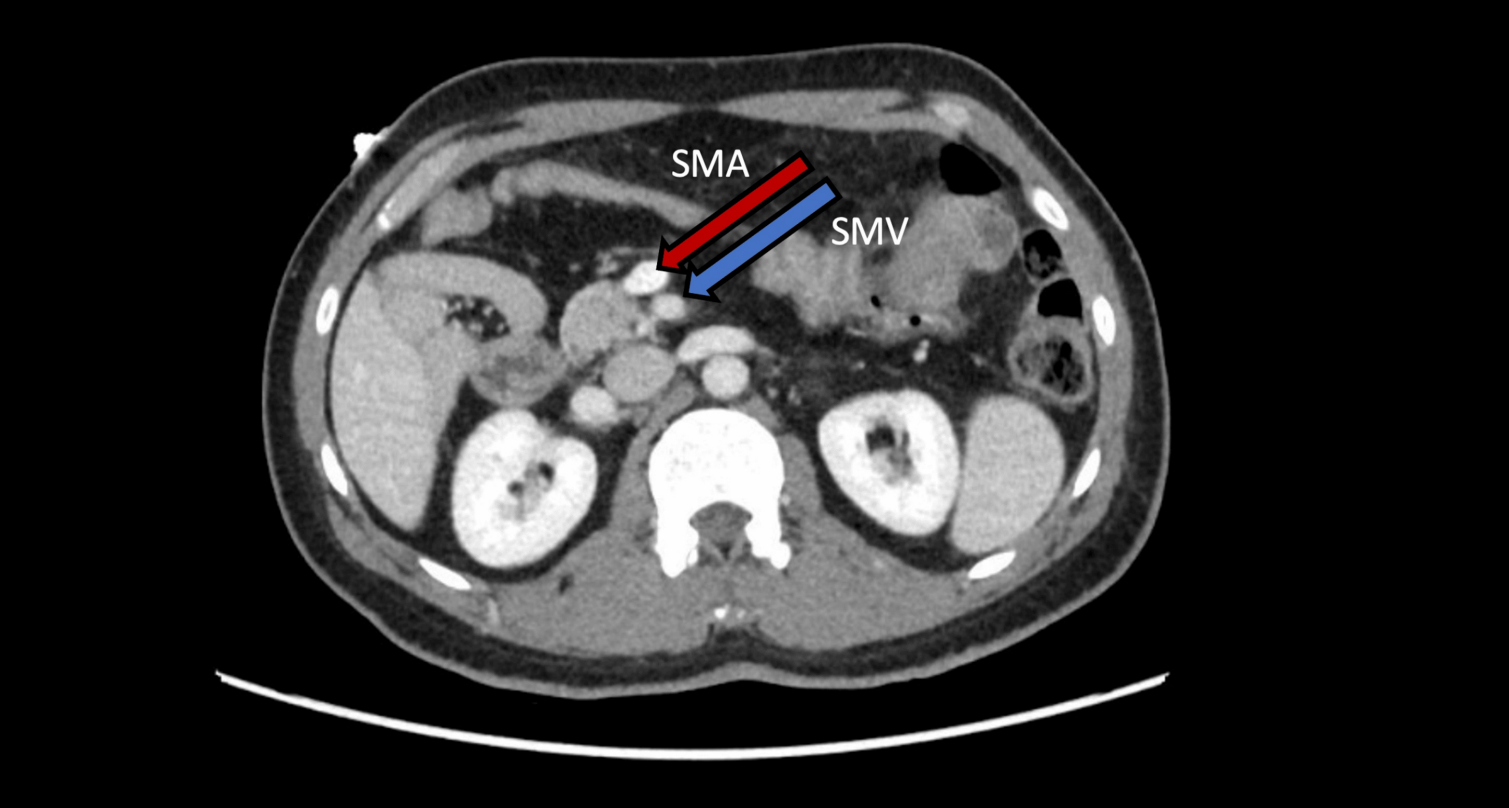
Vertical orientation of the superior mesenteric vessels Axial CT image demonstrating the superior mesenteric artery (SMA) and superior mesenteric vein (SMV) that exhibit a vertical configuration. Under normal anatomy, the SMA lies to the left of the SMV in a horizontal configuration. A vertical SMA-SMV orientation is suggestive of intestinal malrotation. CT, computed tomography.

**Figure 3:**
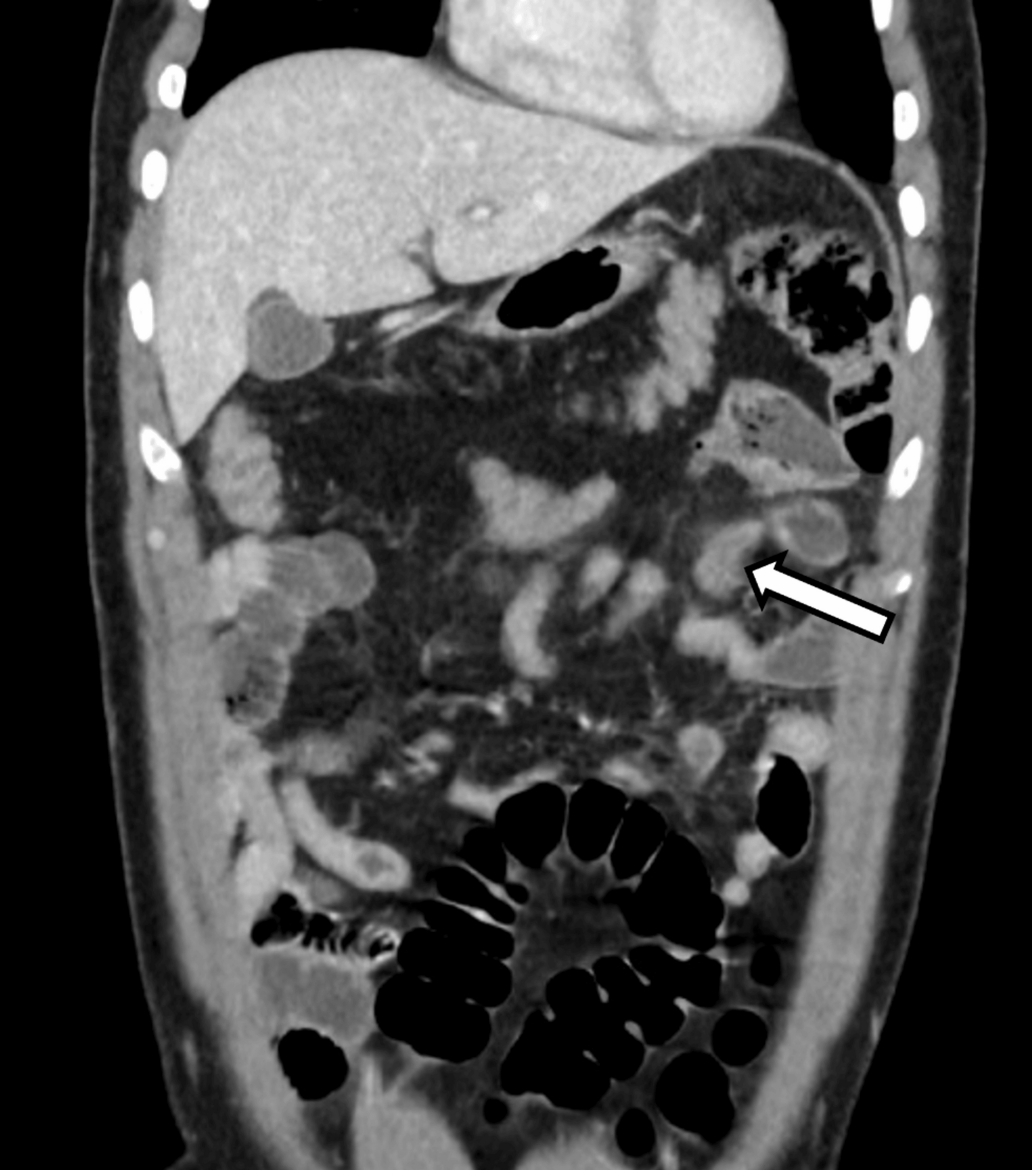
Inflamed appendix in the left upper quadrant Coronal CT image demonstrating the appendix abnormally located in the left upper quadrant, appearing distended with surrounding periappendiceal fat stranding, consistent with acute appendicitis. CT, computed tomography.

A bedside ultrasound of the abdomen performed in the emergency department revealed no hepatobiliary pathology and did not visualize the appendix. Given the patient's clinical picture, further evaluation was performed with a contrast-enhanced computed tomography (CT) scan of the abdomen and pelvis. Imaging demonstrated intestinal malrotation, with failure of the duodenojejunal junction to cross the midline (Figure 1) and a vertical orientation of the superior mesenteric vessels (Figure 2). The cecum and appendix were in the left upper quadrant, anteroinferior to the spleen. The appendix was fluid-filled, distended, with marked periappendiceal fat stranding, consistent with acute appendicitis (Figure 3). No appendicolith, bowel obstruction, midgut volvulus, or manifestations of bowel ischemia were noted.

Differential diagnosis

Given the patient's predominant epigastric pain, vomiting, and fever, initial differentials focused on upper gastrointestinal and hepatobiliary pathology. Gastritis was considered but became unlikely due to symptom progression despite proton pump inhibitor therapy and the presence of systemic inflammatory features. Hepatobiliary causes, including acute cholecystitis and ascending cholangitis, were excluded based on the absence of jaundice, a negative Murphy's sign, normal liver function tests, and unremarkable ultrasound findings. Small bowel obstruction was considered, given the periumbilical abdominal pain, reduced bowel movements, and vomiting. However, the absence of abdominal distension, hyperactive bowel sounds, bilious vomiting, and radiological evidence of dilated bowel loops ruled this out.

Although appendicitis was not suspected initially due to the atypical location of pain, an intra-abdominal source of infection was increasingly suspected as inflammatory markers rose and hemodynamic instability developed. Contrast-enhanced CT imaging ultimately confirmed acute appendicitis occurring in the context of previously undiagnosed intestinal malrotation. Midgut volvulus was assessed due to its known association with malrotation; however, imaging showed no evidence of volvulus or bowel ischemia.

Treatment, outcome, and follow-up

The patient was admitted under the general surgery service following radiological confirmation of acute appendicitis. With a confirmed intra-abdominal source of infection and elevated lactate, he met the Sepsis-3 criteria for sepsis [15]. He was kept nil per os and initiated on empiric intravenous antibiotic therapy with metronidazole 500 mg and piperacillin-tazobactam 4.5 g, every eight hours. Despite initial fluid resuscitation, he remained hypotensive (blood pressure 80/40 mmHg). The intensive care unit (ICU) was urgently consulted, and vasopressor support with phenylephrine was initiated to maintain a mean arterial pressure of ≥65 mmHg, fulfilling Sepsis-3 criteria for septic shock. Following hemodynamic stability, he was taken to the operating theater for an emergency laparoscopic appendectomy.

Given the abnormal anatomy, epigastric and right subcostal ports were used instead of standard port placement. The cecum was identified following the taenia coli, and the inflamed appendix was located in the left upper quadrant. Intestinal malrotation was observed. The base of the appendix demonstrated patchy discoloration but remained viable, with no evidence of perforation or frank necrosis. The appendix was resected and sent for histopathological analysis. 

Following the surgery, the patient was admitted to the ICU in light of persistent hypotension due to septic shock requiring vasopressor support. The phenylephrine infusion was gradually tapered and discontinued, and a norepinephrine infusion was initiated on postoperative day one. After blood cultures confirmed extended-spectrum beta-lactamase bacteremia, antibiotic therapy was escalated to intravenous meropenem 1 g three times daily. 

The patient’s hemodynamic status improved on postoperative day two, and norepinephrine support was gradually discontinued. Antimicrobial therapy was subsequently de-escalated to intravenous ertapenem 1 g once daily, in accordance with culture sensitivities. He remained afebrile, had minimal pain, and tolerated oral fluids. Serial laboratory markers mirrored his ongoing recovery with a decrease of white blood cell count to 7.6 × 10⁹/L, CRP to 44.6 mg/L, and normalization of lactate to 1.3 mmol/L. With continued clinical improvement, the patient was stepped down to the surgical ward. Histopathology results were consistent with acute appendicitis.

The patient made an uncomplicated recovery and was discharged in stable condition on postoperative day six. Outpatient surgical follow-up was advised; however, the patient returned to his home country and elected to continue care there. No further outcome data were available due to this.

## Discussion

Acute appendicitis is a common surgical emergency, with an age-standardized incidence of 229.9 per 100,000 population in 2019, and has a sustained increase worldwide since 1990 [1]. Diagnosis can be challenging when classical clinical features and pain patterns are absent [6,16]. Atypical appendix positioning may result in vague or misleading pain patterns, increasing the risk of diagnostic delay. The absence of right lower quadrant tenderness has been identified as a factor associated with delayed diagnosis of acute appendicitis, spotlighting the limitations of clinical assessment alone in atypical cases [6].

Left-sided or epigastric presentations of acute appendicitis are rare and are most commonly associated with congenital anomalies such as midgut malrotation or situs inversus totalis. In a review of 95 reported cases of left-sided appendicitis, the majority of patients had underlying situs inversus totalis or midgut malrotation, and only 51.5% were diagnosed preoperatively [16]. Acute appendicitis presenting with epigastric pain is even less common, with few case reports describing it [13,17]. Epigastric pain is commonly attributed to cardiac, gastric, hepatobiliary, or pancreatic pathology [18], which can divert attention away from atypical cases of appendicitis. In our patient, this led to an initial misdiagnosis of gastritis. Unlike prior reports of appendicitis presenting with epigastric pain where the appendix was located in the mid-upper abdomen, our patient had a left upper quadrant appendix anteroinferior to the spleen. These atypical presentations stress the importance of considering appendicitis as a differential diagnosis of upper abdominal pain.

Congenital intestinal malrotation is a developmental anomaly resulting from incomplete rotation of the midgut around the superior mesenteric artery between the fifth and twelfth weeks of embryogenesis, leading to abnormal positioning of the cecum and appendix [7]. While it commonly presents in infancy with bilious emesis and acute abdomen, adults are often asymptomatic or experience subtle, chronic, or non-specific symptoms such as abdominal pain and nausea, contributing to delayed recognition [8-12]. In our case, the patient was completely asymptomatic before presentation, and the diagnosis of intestinal malrotation was incidental, identified only through imaging performed for appendicitis.

The World Society of Emergency Surgery recommends a risk-stratified approach to the diagnosis and management of suspected appendicitis, combining clinical assessment, validated scoring systems (such as the Appendicitis Inflammatory Response score or the Adult Appendicitis Score), and laboratory investigations (including white blood cell count and CRP). In adults with intermediate clinical risk, low-dose contrast-enhanced CT is the recommended imaging modality for confirming the diagnosis and reducing the rate of negative appendectomies [19]. CT imaging is especially valuable in cases of appendicitis associated with intestinal malrotation, as it can identify both appendiceal inflammation and underlying anatomical variations. In our patient, contrast-enhanced CT was necessary in localizing the source of abdominal pain and infection, confirming acute appendicitis, and uncovering the varied anatomy. This highlights the importance of early CT imaging in adults with non-classical symptoms, as sole reliance on clinical assessment may lead to delayed diagnosis and an increased risk of complications.

Recognition of the abnormal anatomy facilitated adaptive operative planning, with modification of port placement to accommodate the left-sided appendix. Laparoscopic appendectomy offers flexibility in port positioning, allowing for safe visualization and dissection of the appendix in various positions [20]. Previous documented cases of epigastric or left-sided appendicitis have reported similar tailored techniques in port placement [13,14,17].

A limitation of this report is the lack of long-term follow-up, as the patient returned to his home country after discharge and continued care outside our institution, preventing the assessment of delayed postoperative outcomes. This limitation highlights an important learning point beyond acute care. Identification of anatomical variants has implications for future clinical encounters, particularly if patients present to different healthcare facilities. Clear documentation of intestinal malrotation in discharge summaries may improve continuity of care between facilities, help reduce diagnostic delay, and enhance patient safety.

## Conclusions

Left-sided acute appendicitis secondary to intestinal malrotation is rare and may present with variable pain patterns that obscure diagnosis. This case illustrates the limitations of relying solely on classical clinical algorithms in patients with atypical presentations and emphasizes the need to consider appendicitis in adults presenting with epigastric pain. Familiarity with anatomical variants, early use of CT imaging, and tailored laparoscopic approaches can enable timely diagnosis and safe surgical management. Thorough documentation of anatomical variants in patient discharge summaries is essential for ensuring patient safety and continuity of care, particularly if patients present to other healthcare facilities in the future.
